# On the Use of Manganese as an Adjuvant to Iron

**Published:** 1852-11

**Authors:** 


### RECORD OF MEDICAL SCIENCE.

### MATERIA MEDICA AND THERAPEUTICS.

*On the use of Manganese as an Adjuvant to Iron.*—M. Petrequin quotes various authors to prove that manganese is a normal constituent of animal and vegetable tissues; and believes that wherever iron is present in appreciable quantity, manganese coexists with it. Hence iron alone will not always succeed in blood-diseases. M. Pétrequin has observed many cases of chlorosis, which have resisted iron as obstinately as anæmia connected with cancer or organic degeneration. Other cases again, after deriving a certain amount of benefit from iron, remain stationary. Others again appear cured by iron, but the cure is not permanent. The remedy required in these cases, M. Pétrequin finds to be manganese. He does not give it or iron alone; but combines them.

It is especially in *diseases of the blood* that ferro-manganic medicines are useful. They have a special action on the vascular apparatus, on the formation of the blood, and on the circulating fluid itself. They do not act merely as tonics or astringents; but are regenerators of the blood. They have succeeded admirably in anæmia following hæmorrhage, operations, polypi, metrorrhagia, &c., also in the chlorosis attending puberty, which is a more common disease than is generally supposed, and occurs even in males. M. Pétrequin has also frequently found the combinations of iron with manganese of benefit in the diseases of women at the critical period. He has often seen, in these subjects, *metrorrhagia*, accompanied with an aspect of the surface which would lead to the suspicion of organic uterine disease: the hæmorrhage, however, was but a complication, and the patients, apparently in a hopeless state, have recovered under the use of ferro-manganic preparations, conjoined with tonics and ergotine.

In *amenorrhœa* and *dysmenorrhœa*, the patients often imagine that they require to be bled; but care must generally be taken not to comply with this request. M. Pétrequin has more than once seen cases of amenorrhœa with severe chlorosis, in which it has not been desirable to hasten the appearance of the catamenia—the consequent loss of blood aggravating the disease. The general state of health must here be carefully attended to. Œdema of the lower limbs sometimes occurs in these cases; but it is a less severe complication than when it attends metrorrhagia. It often disappears, as the patient recovers, under the use of iron and manganese.

These medicines are no less efficacious in the treatment of *anæmia* resulting from prolonged intermittent fevers, prolonged suppuration, strumous, syphilitic, or cancerous affections, phthisis, &c. Pills and the syrup of the iodide of manganese and iron are preferable in these cases.

In all these cases, the ferro-manganic preparations do not merely act on the stomach and nervous system, but they are absorbed and assist in the formation of hæmatosine and new blood-globules, so as to restore the blood to its normal condition. Their effect in this way is greater than that of iron alone.

In the *functional affections of the heart* connected with chlorosis and anæmia, and which must not be mistaken for organic disease, a combination of iron and manganese with digitalis and other moderators of the heart's action is advantageous. The same remark applies to the *functional disorders of the lungs*, attending the same constitutional states. *Disordered states of the nervous system* are intimately connected with those of the blood. M. Pétrequin has found that the ferro-manganic preparations succeed well in these, even though uncomplicated with chlorosis. He, as well as M. Gubian, has observed that iron is here better tolerated when combined with manganese. He has also seen benefit from the use of iron with manganese in many cases of *dyspepsia, gastralgia, gastro-enteralgia*. Nervous affections of the digestive organs are often the result of chlorosis; and, where stomachics and cinchona have failed, iron has often been found (especially the carbonate, by some English physicians) to be of service. Gastrodynia complicating chlorosis has often yielded to the use of ferro-manganiferous water, and to pills of carbonate of iron and manganese.

In *nervous affections connected with exhaustion* from venereal excesses, onanism, rapid growth, &c., as well as in leucorrhœa, diabetes, &c., M. Pétrequin has a high opinion of these medicines. He is continuing his researches on their action in certain cases of sterility from asthenia and in some hyposthenic affections of the scalp, such as early baldness, alopecia, &c.

M. Pétrequin has confined his observations to a limited number of the ferro-manganic preparations; and has made many observations before publishing the formulæ which he finds most useful. Having found, even at an early period, that the medicines were liable to adulteration, he has availed himself of the assistance of competent pharmaceutists. Since the publication of his first memoir, in 1849, these medicines have been extensively used in the south of France and in foreign countries.

The formulæ are few and correspond to the preparations of iron generally used in France. They are; 1. *Pills* of carbonate of iron and manganese, or of iodide; 2. *Lozenges* of lactate of iron and manganese; 3. *Syrups* of lactate or of iodide of iron and manganese; 4. Ferro-manganic *chocolate;* 5. *Effervescing solution* of iron and manganese.

It has been observed that manganese not only preserves water, but purifies that which has undergone change—(Martin-Lauzer.) Ferro-manganic waters (of which there are many in France and other parts of the continent) can be preserved and carried to a distance;—which cannot generally be done with simple ferruginous waters.

M. Pétrequin commences by giving a powder of iron and manganese, with some vinous drink; he then administers two pills daily, one before breakfast and one before dinner, replacing them soon by the lozenges. The syrups and chocolate complete the treatment. He gives the medicines at meal time. The syrup he gives before breakfast, in doses of a teaspoonful; and he finds it useful to administer directly after it some infusion of centaury, or of chamomile flowers and orange.

Large doses are unnecessary and useless; for they are liable to produce irritation of the stomach and exhaustion of the nervous system; and the reparation of the blood is slow and progressive, and cannot, even were it desirable, be effected rapidly. Besides the iron and manganese are not absorbed in any greater quantity, if large doses are given.—*Dublin Med. Press.*

